# Is blood cortisol or vateritic otolith composition associated with natal dispersal or reproductive performance on the spawning grounds of straying and homing hatchery-produced chum salmon (*Oncorhynchus keta*) in Southeast Alaska?

**DOI:** 10.1242/bio.042853

**Published:** 2019-06-10

**Authors:** Casey J. McConnell, Shannon Atkinson, Dion Oxman, Peter A. H. Westley

**Affiliations:** 1 College of Fisheries and Ocean Sciences, University of Alaska Fairbanks, 323 Garteeni Hwy, Hoonah, AK 99829, USA; 2 College of Fisheries and Ocean Sciences, University of Alaska Fairbanks, 17101 Lena Point Loop Road, Juneau, AK 99801, USA; 3 Alaska Department of Fish and Game, 10107 Bentwood Pl, Juneau, AK 9901, USA; 4 College of Fisheries and Ocean Sciences, University of Alaska Fairbanks, 905. N Koyukuk Drive, Fairbanks, AK 99775, USA

**Keywords:** Stress, Straying, Vaterite, Cortisol, Hatchery, Salmon

## Abstract

Homing with high fidelity to natal spawning grounds for reproduction is a hallmark of anadromous Pacific salmon biology, although low rates of dispersal (‘straying’) also occurs. Currently little is known about the proximate factors influencing straying, which limits our understanding of this fundamental biological phenomenon and impedes options for reducing straying-mediated interactions between wild and hatchery-produced individuals. We explored the potential role of stress experienced in captivity prior to intentional release to manifest in developmental irregularities and potentially influence rates of straying by adults. We compared two proxies for stress between groups of hatchery-produced individuals that had homed back to the hatchery or strayed to non-natal streams compared to wild individuals that were presumed to have homed to a wild spawning stream. Blood plasma cortisol was used to assess stress at the terminus of their migration, and percent frequency of vateritic otolith development within groups as a measure of stresses incurred during development. We found no evidence that either proxy for stress was associated with straying. No differences in cortisol concentrations were found between wild and hatchery-produced chum salmon that had homed or strayed, either in males (wild=95.9±175.7 ng/ml; stray=113.4±99.7 ng/ml; home=124.7±113.8 ng/ml) or females (wild=307.6±83.4 ng/ml; stray= 329.0±208.9 ng/ml; home=294.1±134.8 ng/ml); however, significant differences between males and females occurred in each group. The percent frequency of vaterite occurrence in otoliths of hatchery-produced chum salmon that either strayed (40% vaterite) or homed (45% vaterite) did not differ significantly, though rates of vaterite occurred less frequently in wild chum salmon (24%), which is consistent with other studies. Mass thermal marking of juvenile fish in hatcheries is unlikely to increase vateritic development as neither intensity (number of temperature changes) or complexity (number of temperature change sequences) of the mark was associated with frequency of vaterite occurrence. Though not associated with straying, cortisol concentrations were associated with shorter instream lifespan of both hatchery and wild individuals but did not appear to influence rates of egg retention in spawning females, suggesting an equivocal role in reproductive ecology. Our results are suggestive that stress induced during the early stages of rearing in a hatchery environment from marking or other causes may not increase straying later in life, though the higher rates of vaterite observed in hatchery-produced fish may come at a cost of increased marine mortality, due to the otoliths' role in navigation and hearing.

## Abstract (precis)

**Summary:** Straying mediates ecological interactions and gene flow between salmon populations. Understanding physiological controls and underlying causes of straying by hatchery-produced salmon may help managers minimize deleterious interactions.

## INTRODUCTION

Homing of Pacific salmon (*Oncorhynchus* spp.) to natal sites for reproduction is entwined with the ecology, evolution and management of the species (Myers et al., 1998; Dittman and Quinn, 1996). The alternative to homing, termed ‘straying’, has garnered much attention because it facilitates (re)colonization of potentially accessible habitats (e.g. Pess et al. 2012; Keefer and Caudill, 2014) and mediates interactions between hatchery and wild fish on the spawning grounds (Quinn, 1993; Rand et al., 2012). Although it is clear that straying is associated with phenotypic attributes of dispersers such as size or sex (Lin et al., 2008) and is plastic in response to climatic factors (Westley et al. 2015), density dependence (Berdahl et al., 2016), and anthropogenic disturbance during juvenile imprinting (Keefer et al., 2008; Bond et al., 2017), the proximate physiological factors associated with straying are not well known (reviewed in Dittman and Quinn, 1996). Physiology and behavior of a wide range of taxa are mediated by hormone production and monitoring those hormone levels are often useful in understanding life history variations within species (Crespi et al., 2013).

Baseline activity of the hypothalamo-pituitary-adrenal axis (HPA) which produces glucocorticoid hormones (including cortisol) generally regulates homeostasis and HPA activity can increase naturally during certain life-history stages (McEwen and Wingfield, 2003). Stress-inducing situations stimulate the HPA to increase production of glucocorticoid's to meet energetic demands of perceived challenges to homeostasis (Wingfield and Sapolsky, 2003). This temporary increase in hormone production above basal levels is commonly measured as an acute stress response and used in models as an explanatory variable to predict various life history and behavioral responses (Crespi et al., 2013).

Among salmonids, glucocorticoid's – and cortisol in particular – serve a fundamental role in the parr-smolt transformation (Langhorne and Simpson, 1986; Iwata, 1995) and the homeward migration to spawning grounds by adults (McBride et al., 1986). Elevated concentrations of circulating cortisol (hereafter referred to simply as ‘cortisol’) were observed in upstream migrating adult kokanee (*Oncorhynchus* *nerka*) even though their migration was not especially rigorous and lacked salinity changes associated with stress responses in salmon migrating from a marine environment (Carruth et al., 2000a). It is unclear how the potential benefits of elevated cortisol involved with homing are balanced with the detrimental impacts of chronically high cortisol levels, which can alter maturation rates, reduce immune capacity, accelerate senescence, and contribute to pre-spawn mortality (Schreck et al., 2001; Hruska et al., 2010; Cook et al., 2011; McConnachie et al., 2012). The HPA in salmon also appears to be involved in olfactory recognition as regions of the salmon brain associated with memory and olfaction are sensitive to corticosteroids (Carruth et al., 2000b), which may help to assist with natal homing (Dittman, 1994). The elevation of cortisol concentrations during the homeward migration phase could be an adaptive mechanism to enhance recall of learned natal odors, which assists with successful navigation to home streams (Hasler and Scholz, 1983; Dickhoff, 1989; Dittman, 1994; Carruth et al., 2002). Cortisol concentrations may vary among individuals in response to navigational challenges during homeward migrations, predator avoidance, or thermal stress, and may combine to influence fitness (Wendelaar-Bonga, 1997; McConnachie et al., 2012). Thus, differences in cortisol concentrations may be expected between groups that successfully home and those that stray to non-natal locations. If the physiological response that leads to correct identification of natal source is significantly different from the response that causes homing failure, there may be cascading effects of physiological differences between homing and straying salmon once they reach their spawning grounds. A difference in levels of a stress response, measured through changes in cortisol concentrations, may further help to explain why strays typically display lower reproductive success than salmon that have correctly homed, beyond typical local adaptations and phenotypic differences (Peterson et al., 2014).

As in other taxa (reviewed by Palmer, 1994), chronic stress during early development of salmonids can be manifested in easily identifiable bioindicators of otherwise difficult to measure developmental abnormalities (Palmer and Strobeck, 1986; Campbell, 2003). Specifically, evaluating developmental disruption, from either extrinsic (environmental variation) or intrinsic (e.g. outbreeding depression via disruption of co-adapted gene complexes) sources, is commonly quantified in fishes by comparing differences in counts of paired fin rays, branchiostegal rays, maxillary length and gill rakers (Bryden and Heath, 2000; Dann et al., 2010) as well as differences in otolith size, shape and composition, i.e. vaterite deposition (Oxman et al., 2005; Díaz-Gil et al., 2015). Otoliths are typically formed of aragonite, though they may occasionally be composed of vaterite, a polymorph of calcium carbonate that appears clear and is commonly described as crystalline (Campana, 1999). The mechanistic cause of aragonite versus vaterite deposition is unknown, though hatchery-produced salmon consistently show higher rates of vaterite compared to their wild counterparts (Sweeting et al., 2004; Bowen et al., 2011; Reimer et al., 2016). Factors that may lead to higher rates of vateritic formation in hatchery individuals may include crowding, rapid growth, noise, or mechanical shock that occurs within hatcheries, all of which can be considered potential stressors (Strong et al., 1986; Sweeting et al., 2004; Fagerlund et al., 2011; Reimer et al., 2017). Temperature fluctuation is another within-hatchery stressor hypothesized to be associated with the occurrence of vaterite (Oxman et al., 2005).

**Fig. 1.**
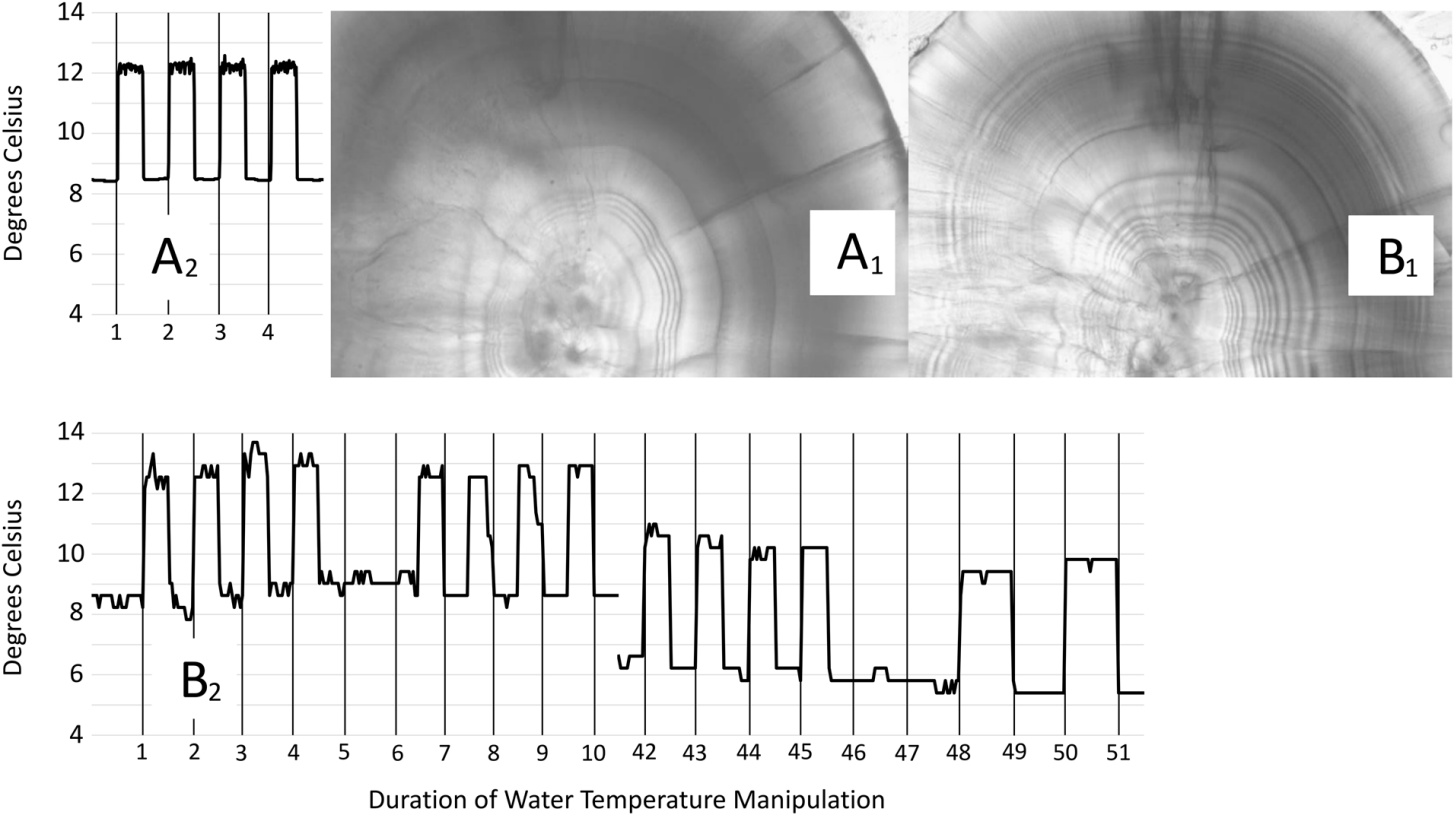
**Thermal profiles experienced by pre- and post-hatch chum salmon.** Fine vertical bars on graphs represent the ending of a 24-h period (not necessarily at 0:00). Inset images ‘A1’ and ‘B1’ are otoliths from alevin exposed to the corresponding thermal patterns. ‘A2’ mark is a low intensity, low complexity mark designated *4H*, whereas ‘B2’ is a high intensity and high complexity mark designated *4,4H4n,2*. Note jump in x-axis scale of profile ‘B’ where a 32-day pause in marking occurred to allow for hatching of embryos.

During the embryonic and larval stages, virtually all chum salmon released each year into Alaska's waters are intentionally exposed to abrupt water temperature changes (typically 3–4°C) that disrupt natural growth leaving distinct visible marks within the otolith (Volk et al., 1999). This process of ‘thermal marking’ serves to mass mark individuals with unique codes that allow for identification of origin, release location and age (see Fig. 1). Thermal marking events can last days or weeks. The number of rings and spacing of cycles in thermal marks vary considerably (purposefully) to increase distinction between mark patterns to aid in identification by fishery managers (Volk et al., 1999). For example, a more complex mark would have more temperature fluctuations, take longer to apply and may have multiple iterations of mark application, resulting in a longer period of potential stress for the embryo or alevin. If stress-induced developmental disruptions coincide with the olfactory imprinting process, then the ability to home accurately may be compromised by such marking, which could prove to be problematic for managers who rely on thermal marks to differentiate between hatchery and wild fish as part of their management conservation efforts. To date, the potential impact of hatchery-induced stress on homing abilities of chum salmon remains unknown.

We explored how stress imparted at the beginning and end of the chum salmon life cycle might disrupt physiological development, migratory behavior and influence reproductive performance on the spawning grounds. Through paired comparisons between wild fish born in nature that were presumed to have homed to natal areas, hatchery-produced fish that were known to have strayed onto wild spawning grounds, and hatchery-produced fish that returned home successfully, we quantified blood plasma cortisol concentrations and its association with two traits associated with reproductive success: instream lifespan and egg retention. Additionally, we compared the frequency of vateritic otolith crystallization between straying and homing salmon to determine if thermal fluctuations experienced during early development in captivity is correlated with straying later in life. To the extent that stress may influence homing and straying, we expected that (1) hatchery-produced strays would have higher cortisol concentrations compared to either naturally-produced or hatchery-produced chum salmon that homed successfully, (2) individuals with higher cortisol concentrations would have shorter instream lifespans and display higher rates of egg retention than individuals with lower cortisol concentrations, (3) frequency of vaterite occurrence would increase across a gradient of potential stress as thermal mark intensity and complexity increases, and (4) that hatchery-produced strays would have higher frequency of vaterite occurrence compared to hatchery-produced fish that homed successfully.

## RESULTS

### Frequency of vaterite occurrence, straying and thermal mark intensity

Thermal marking was not associated with percent frequency of vaterite occurrence regardless of thermal marking intensity (z=1.27, *P*=0.203), complexity (z=1.27, *P*=0.259), or duration (z=1.36, *P*=0.172) between different hatchery groups of chum salmon from SSRAA. Groups from SSRAA had 32%, 32% and 39% frequency of vaterite occurrence (Table 1). The frequency of vaterite occurrence in ‘naturally-produced’ (Np) chum salmon in Sawmill Creek was 24%, which was lower than any of the hatchery-produced (Hp) groups from DIPAC or SSRAA (Table 1). Furthermore, vaterite occurrence was not associated with straying. Rates of vaterite occurrence did not differ between hatchery-produced strays (Hps) (40% vaterite) and hatchery-produced home (Hph) (45% vaterite) chum salmon (Z-test; Z score=1.22, *P*=0.222) despite our ability to control for brood-year effects and using groups with large sample sizes (Table 1).

**Table 1.**
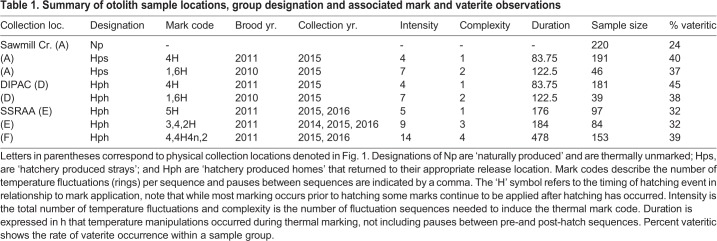
**Summary of otolith sample locations, group designation and associated mark and vaterite observations**

### Straying, lifespan, egg retention and cortisol

**Fig. 2.**
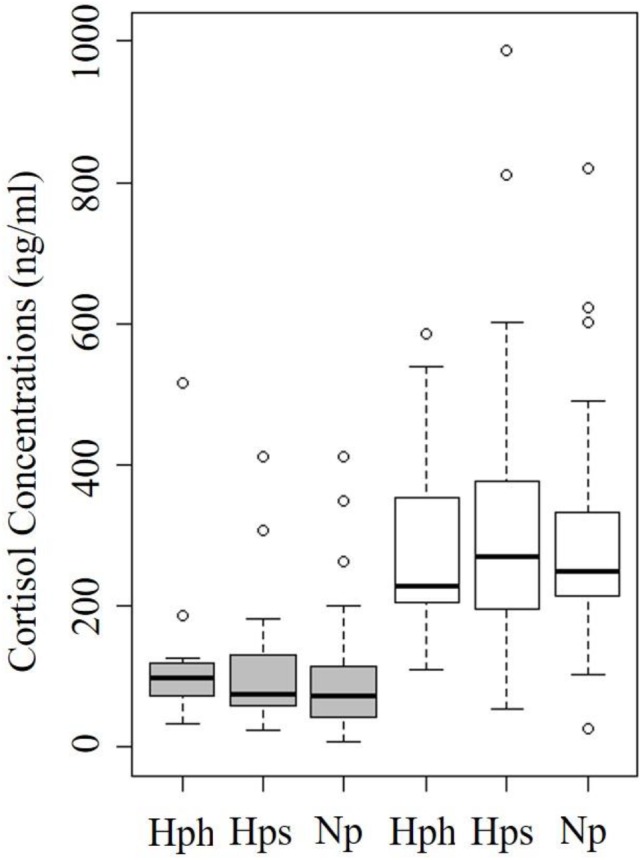
**Cortisol concentrations in ng/ml of Hph, Hps and Np groups.** Shaded boxes represent values of male chum salmon and white boxes represent values of female chum salmon.

Male chum salmon had significantly lower cortisol concentrations (104.9±92.9 ng/ml) than female (319.9±176.9 ng/ml) chum salmon (GLM; z-value=−6.43, *P*<0.001) so comparisons were conducted separately for each sex. We found no evidence that cortisol was associated with straying in either males or females (Fig. 2). Cortisol concentrations from 15 Hph males in Salmon Creek (mean: 124.6±113.8, range: 32.7–516 ng/ml) did not differ significantly (GLM; t-value=−0.55, *P*=0.57) from the 18 Hps males collected in Sawmill Creek (mean: 113.4±99.7, range: 23.0–412 ng/ml). Twenty-five Hph females were sampled at Salmon Creek and their cortisol concentrations (mean: 294.1±134.8, range: 109.6–586 ng/ml) were also not significantly different (GLM; t-value=0.22, *P*=0.82) than the 31 Hps females collected from Sawmill Creek (mean: 329.0±208.9, range: 55–988 ng/ml). Cortisol concentrations were slightly lower in the 43 Np males (mean: 95±83.7, range: 7.2–412 ng/ml) than the pooled Hph and Hps males though it was not a significant difference at the specified alpha level; (GLM; t-value=−1.72, *P*<0.089). A significant difference was not found between pooled Hph and Hps females and 24 Np females (mean: 307.6±175.7, range: 25.6–820 ng/ml; mean: 95±83.7, range: 7.2–412 ng/ml; GLM; *t*-value −0.33, *P*=0.74).

**Fig. 3.**
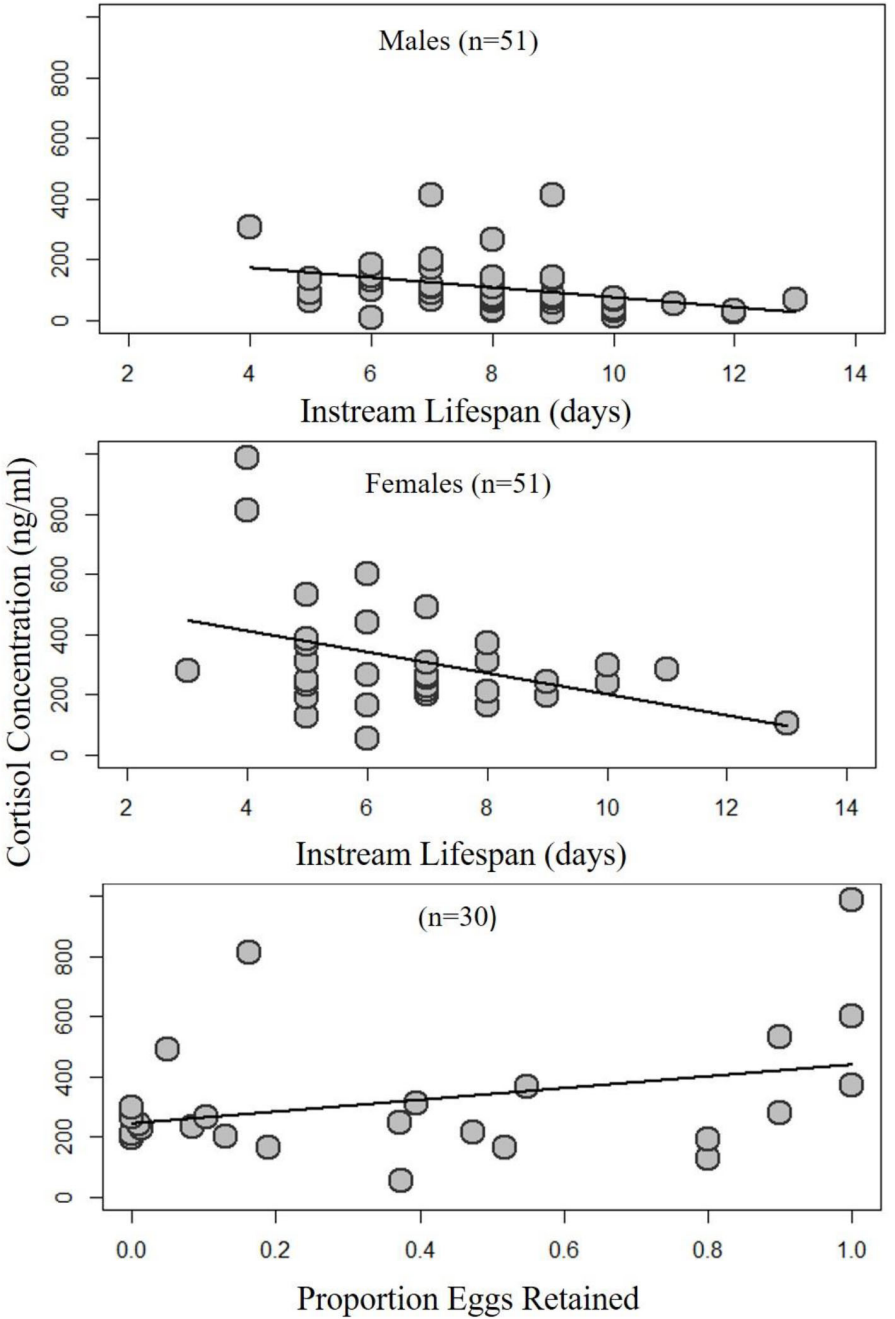
**Normal otolith comprised of aragonite (A) and otoliths with aragonite core that partially transitioned to vaterite (B,C).** Dashed lines in panels B and C detail transition zone and the vateritic portion is denoted by the * symbol. Otoliths were photographed sulcus side down under dissecting microscope using double polarizing filters and projected light.

While there were no differences between Np and Hps, higher cortisol concentrations were associated with shorter instream lifespan in both the 51 males (GLM; *t*=−3.44, *P*=0.001) and 40 females (GLM; *t*=−2.65, *P*=0.01; Fig. 3). In contrast, the levels of egg retention of 30 females that died of natural causes was not significantly related to their cortisol concentrations (GLM; z=−1.06, *P*=0.28; Fig. 3). No other biological or ecological measures were significantly correlated with cortisol concentrations, except for a statistically significant positive relationship with temperature among male chum salmon (Table 2).

**Table 2.**
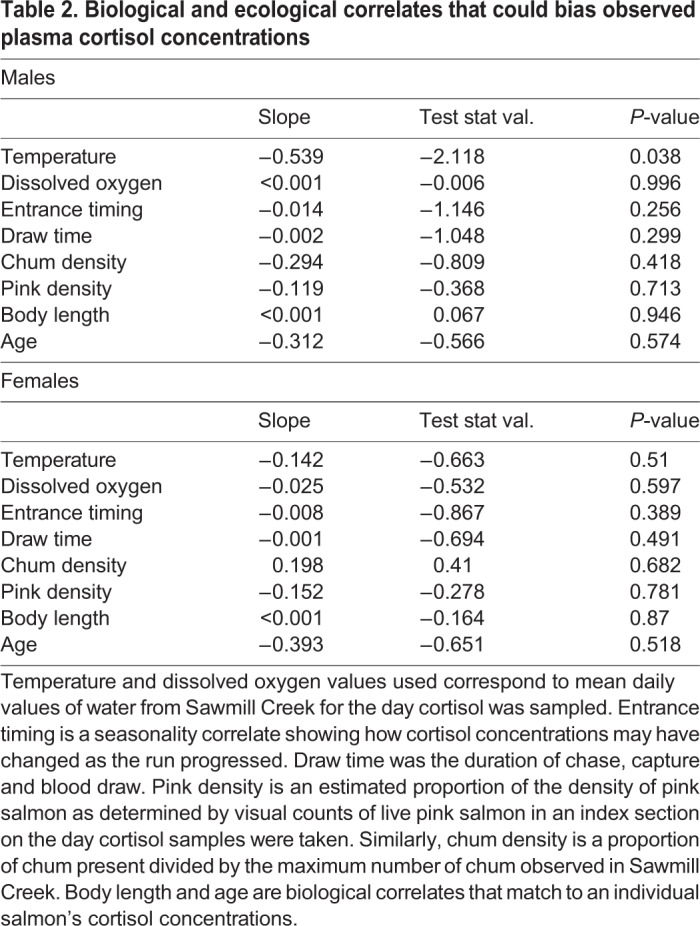
**Biological and ecological correlates that could bias observed plasma cortisol concentrations**

## DISCUSSION

We found no evidence that cortisol concentrations in blood plasma in adults returning to freshwater or frequency of vaterite occurrence in otoliths was associated with straying in hatchery-produced chum salmon in Southeast Alaska. Moreover, we detected no effects of intensity, complexity, or duration of thermal marking on the likelihood of straying. We could not, however, determine if thermal marking per se influenced straying because we had no unmarked groups to serve as controls. Although our putative metrics of stress were not associated with straying we are mindful that stress as a whole may still play a role in dispersal decisions. We observed that stream life – an important proxy for reproductive performance – declined with increasing cortisol concentrations but that the likelihood of prespawning mortality resulting in egg retention in females did not. Consistent with existing literature (Sweeting et al., 2004; Reimer et al., 2016), we observed that hatchery-produced fish had a higher incidence of vateritic otoliths than naturally-produced fish, though the mechanism leading to increased vaterite or the consequences for survival are unclear. Taken as a whole, these results provide evidence that straying may not be directly affected by stress as measured by proxies of vaterite or cortisol concentrations and thus management or hatchery practices designed to lower stress with the goal of reducing straying are unlikely to have an effect.

### Cortisol and straying

We attempted to control for factors known to increase basal cortisol levels by choosing a system that was very short and had only one spawning channel, so that we were assured all salmon sampled would have very similar migration distance, and would theoretically enter the stream (and be sampled) at the same maturity level. This approach had trade-offs, as it was likely that osmoregulatory changes that also effect cortisol levels may have been happening at the same time as the fish were being sampled.

While the majority of cortisol concentrations measured in homing and straying chum salmon fell within the reported range of concentrations for sexually maturing salmonids (Pottinger et al., 1995; Carruth et al., 2000a), the variation associated with these measurements was large, sometimes exceeding those reported for juvenile Chinook salmon (*Oncorhynchus* *tshawytscha*) after exposure to acute stress. Variances in cortisol concentrations observed in this study exceeded those reported by Hruska et al. (2010) and Cook et al. (2011), although those studies were for different salmon species. Some degree of variation in plasma cortisol concentrations are expected as salmon physiology continuously responds to changes in osmoregulation, maturation, spawning activity, senescence and environmental conditions (McConnachie et al., 2012). Given that the variation in our samples were sometimes greater than previously reported for other salmon species, it is possible that some aspect of our sampling design or the surrounding environment led to increased variation in the metabolic response of the chum salmon. For example, we sampled fish at the marine/freshwater interface where presumably physiological transitions were occurring, in contrast to fish from other studies that were sampled far from the marine environment and after completion of osmoregulatory transitions (Wendelaar-Bonga, 1997; Hruska et al., 2010; Cook et al., 2011).

Alternatively, the high variance in cortisol concentrations may have reflected recent encounters with predators, such as harbor seals (*Phoca vitulina*) and Steller sea lions (*Eumetopias jubatus*), which were occasionally observed in the nearshore waters (C. McConnell personal observations). Ultimately the causes of the large variation in cortisol are unknown, but are likely due to the sampling occurring at the interface of the marine and freshwater margin. Nonetheless it may make detection of any true signal of cortisol on straying much more difficult to detect without a greatly increased sample size or a way to incorporate the varying degrees of osmotic balance that are occurring.

Despite the overall large variation in cortisol concentrations, male chum salmon in Sawmill and Salmon Creek exhibited significantly lower cortisol concentrations than females. This contradicts Fagerlund (1967) and Cook et al. (2014) who found no difference between sexes in sockeye salmon. However, higher cortisol in females was also reported by Kobokawa et al. (1999) and Cook et al. (2011) who found both male sockeye and pink (*Oncorhynchus* *gorbuscha*) salmon had significantly lower basal cortisol concentrations than conspecific females. The lower concentration of cortisol concentrations found in males may be in part explained by the timing of our sampling of individuals, which occurred prior to their onset of competitive interactions with other males for access to spawning females. These interactions could elicit chronic stress responses increasing cortisol levels and confounding our ability to test hypotheses regarding links between cortisol and behaviors (straying or homing) that happened prior to spawning that could have influenced homing abilities. Relatively high female cortisol concentrations may reflect the final processes of ova development (Pickering and Pottinger, 1987) or increased activity levels while egg-laying but are unlikely to reflect sex-specific run timing as cortisol concentrations did not vary systematically throughout the season (from exploratory analysis of entrance timing, see Table 2).

**Fig. 4.**
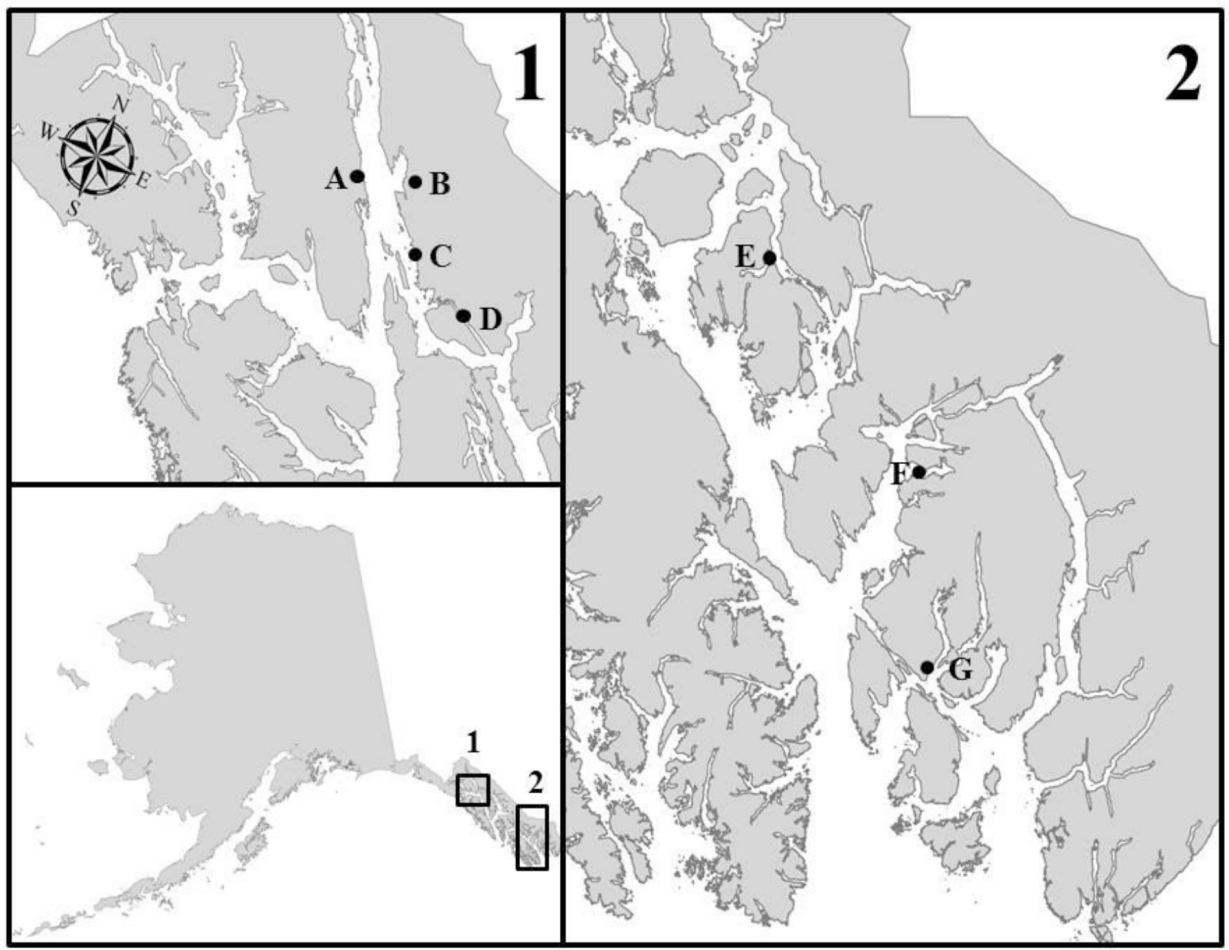
**Map of study area showing hatchery, release and sampling locations.** (A) Douglas Island Pink and Chum Hatchery (DIPAC) remote release location at Boat Harbor, (B) Sawmill Creek, (C) DIPAC remote release location at Amalga Harbor, (D) Salmon Creek and DIPAC facility where egg collection and rearing occurs, (E) Anita Bay Terminal Harvest Area, (F) Southern Southeast Regional Aquaculture Association (SSRAA) rearing facility and release location in Neets Bay, (G) SSRAA Whitman Lake rearing facility.

Although previous evidence suggests that elevated cortisol concentrations might be associated with home stream odor recall during migration (Carruth et al., 2000b) we found no evidence that cortisol concentrations differed between fish that homed or strayed, nor between hatchery-produced and Np individuals. It is possible that Hps entering Sawmill Creek could have initially homed successfully to their imprinted release location, found no suitable spawning habitat and left the area in search of suitable streams, thereby exhibiting a physiological response similar to that of fish that homed correctly. This interpretation has some merit, as (1) the nearest release location at Boat Harbor (ca. 25 km to the northwest) contains only one small creek that is not identified as chum salmon spawning habitat and (2) for the majority of the summer a weir is constructed on the freshwater source of Amalga Harbor (second nearest release location to Sawmill Creek; Fig. 4), preventing the entry of returning hatchery fish (Alaska Department of Fish and Game anadromous waters catalog: https://www.adfg.alaska.gov/sf/SARR/AWC/, accessed June 2016, A. Zaleski, Douglas Island Pink and Chum, Inc. Juneau, Alaska; personal communication, December 2016). The fact that hatchery-produced strays were observed in Sawmill Creek significantly later in the season than Np fish tends to support our hypothesis that hatchery-produced fish could have first returned correctly to their natal release locations, then ‘decided’ to stray upon failing to find suitable freshwater habitat in those locations.

Although our hypotheses regarding cortisol concentrations of home and stray groups of salmon were not supported, the collection of these data did allow for an examination of cortisol's role on instream lifespan and egg retention. We found that increased cortisol was associated with decreased lifespan in both sexes. This is consistent with existing literature regarding cortisol concentrations and salmon lifespans on the spawning grounds (Cook et al., 2011; McConnachie et al., 2012). Egg retention rates, however, did not change with increasing cortisol concentrations. Curiously, Hp strays retained nearly twice as many eggs as Np individuals (McConnell et al., 2018) and the proximate cause of this was not reflected by cortisol concentrations. These results do not indicate a clear role of stress in explaining reductions in relative reproductive success that have been shown between immigrants (either wild or hatchery) and local fish on the spawning grounds (Chilcote et al., 1986; McGinnity et al., 2003; Hendry, 2004).

### Vaterite and straying

Similar to cortisol concentrations of homing or straying chum salmon, the occurrence of vateritic otoliths was not significantly different between groups. This suggests that (1) the mechanism that initiated the switch from deposition of aragonite to vaterite was not positively correlated to straying later in life and (2) the functional impact of an abnormally formed otolith may have had no influence on homing ability, or at least did not supersede other senses such as olfaction or reliance on behaviors like schooling.

**Fig. 5.**
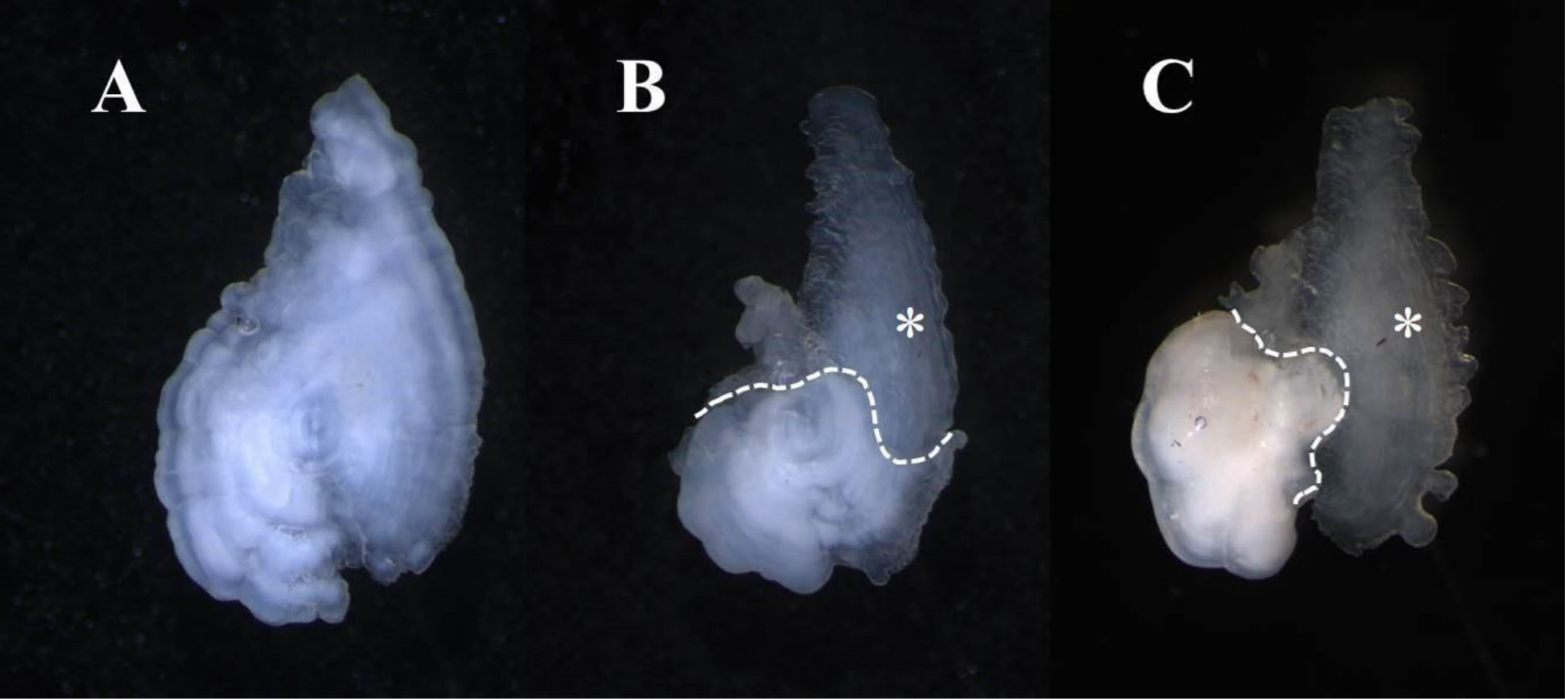
**Normal otolith comprised of aragonite (A) and otoliths with aragonite core that partially transitioned to vaterite (B,C).** Dashed lines in panels B and C detail transition zone and the vateritic portion is denoted by the * symbol. Otoliths were photographed sulcus side down under a dissecting microscope using double-polarizing filters and projected light.

We found no positive evidence that the degree of thermal marking in hatcheries is associated with vaterite occurrence. Unfortunately, because we could not assess stray rates and vateritic otoliths of unmarked hatchery fish (all hatchery fish are marked) we were unable to determine if thermal marking itself was correlated to vaterite production or straying. It remains unclear whether the thermal marking procedure takes place during a critical imprinting period for chum salmon, avoiding disruption during the development processes surrounding olfactory imprinting (Dittman and Quinn, 1996; Ueda et al., 2015). However, given that many otoliths inspected in this study transitioned from aragonite to vaterite prior to their first winter (see Fig. 5, panels B and C) rather than near or at the primordia (very early age) it suggests that thermal marking, which is conducted on embryos, probably had little influence on the production of vaterite or on straying in adults.

We initially hypothesized that the frequently observed switching of aragonite to vaterite near the core of Hp salmon might due to the forced transition to marine conditions and disruption of growth. Forced transitions would theoretically introduce changes in osmoregulation, temperature and foraging more abruptly than if transitions were volitional, though wild salmon can also display smoltification checks in their otoliths and scales that correspond to disrupted and slower growth during the transition to saline environment (Vega et al., 2017), suggesting that even volitional transitions can be stressful. A recent study by Reimer et al. (2017) found higher prevalence of vaterite in groups of salmon that were reared at higher temperatures, suggesting that growth rates may be a cause of vaterite formation. This suggests the cause of switching from aragonite to vaterite around the transition area we observed may be attributable to accelerated growth after release into a marine environment rather than a disruption in growth. Because we did not specifically count daily growth rings, matched to known hatchery release days and correlated to aragonite to vaterite transition zones, we cannot definitively say that it is a release effect, though distilling cause and effect to a single source may be overly simplistic given the variety of stressors and possibility of interaction effects between them.

We found no evidence linking vaterite occurrence to the extent of thermal marking, assessed by levels of intensity, complexity, or duration of the thermal marking procedure. Due to our reliance on archived otolith samples, we were limited to measuring vaterite presence as a potential indicator of developmental instability, whereas comparison of a suite of responses may have yielded different interpretations (Palmer and Strobeck, 2001; Campbell, 2003; Oxman et al., 2013). Curiously, post-hatch chum salmon that had been thermally marked during hatching had higher cortisol concentration levels than control groups (Sanders, 2012). Although the results of Sanders (2012) support the inferred mechanism for thermal marking (i.e. minute growth disruptions caused by short-lived thermal stress), it is not known if this potential stressor influences the development of other sensory organs or has other lasting effects on the physiology of thermally marked fish.

Despite the lack of association between vaterite and straying, we observed that wild chum salmon had consistently lower rates of vaterite occurrence (24% vateritic) than hatchery-produced individuals that had strayed into Sawmill Creek (40% vateritic), which is consistent with previous literature on hatchery and wild salmon otolith composition (Sweeting et al., 2004; Reimer et al., 2016). Although some aspect(s) of rearing within the hatchery environment clearly influences rates of vaterite formation (Reimer et al., 2017), our results suggest that it is not directly related to thermal marking procedures. Also, Hps and Hph had similar rates of vaterite occurrence, indicating that any potential link between vaterite occurrence within a population and that population's propensity to stray are not direct. The role of accelerated growth in Hp fish is a parsimonious explanation and worthy of future work. Because vaterite is known to inhibit auditory function (Oxman et al., 2007) and may result in increased mortality at sea, there may be a trade-off between the survival benefit gained by accelerated growth and increased smolt size versus the mortality incurred by increased vaterite. Quantifying this tradeoff is a potential avenue for future work with fundamental and applied management consequences.

## MATERIALS AND METHODS

### Study site and experimental groups

Sawmill Creek is a small (<1 km length) remote creek in Southeast Alaska (Fig. 4) that supports a wild spawning population of chum salmon. It is located near two release locations used by Douglas Island Pink and Chum hatchery (DIPAC, Juneau Alaska), and is known to routinely attract a substantial number of hatchery-produced strays that are not harvested in nearby common property commercial fisheries (Piston and Heinl, 2012; McConnell et al., 2018). Because of its high rates of recipient straying (78% in 2009 and 47% in 2010; Piston and Heinl, 2012) and relatively small naturally occurring population of chum salmon, Sawmill Creek offered an opportunity to test hypotheses related to straying and homing within the contexts of physiology and ecology. Furthermore, Sawmill Creek was an ideal location because it has been known to contain strays from DIPAC that are reliably identified due to 100% marking at the hatchery and an existing mark reading protocol (Scott et al., 2001).

We defined fish as Hph if individuals were collected as adults at their original release location (either the hatchery facility or a remote-release location). These individuals were confirmed ‘home’ if they possessed the correct thermal mark, as thermal mark patterns are unique among hatcheries and release locations. Hatchery-produced strays were thermally marked adults found in freshwater systems that were not the location of their release or immediately adjacent to their location of release. Salmon without thermal marks that were captured in freshwater were assumed to have correctly homed to their natal stream and defined as Np; however, it is possible that an unknown fraction of unmarked fish could also have been wild strays from other streams. Individuals representative of Np and Hps groups were collected in Sawmill Creek, whereas Hph samples came from either Salmon Creek (adjacent to and water source of DIPAC), the raceway at DIPAC itself, or from Southern Southeast Regional Aquaculture Association (SSRAA) release sites (Fig. 4). Otoliths collected at hatchery facilities were extracted by staff to assess age structure of returning brood and archived after use.

### Analysis of vaterite and associations with thermal mark intensity

Otoliths used in this study for vaterite comparisons were representative of the greatest degree of difference between thermal marks applied to chum salmon in Alaska, and those not directly collected by authors in Sawmill Creek and at DIPAC were donated from archived stores. We applied the terms intensity and complexity to characterize the amount of potential stress experienced by the fish during the marking processes. Mark intensity was defined as the number of thermal cycles induced during the thermal marking process. Mark complexity was a categorical variable based on the number of thermal marking sequences. For example, a thermal mark designation of 4H would have undergone four thermal cycles prior to hatching (denoted by ‘H’), but only one sequence of thermal cycles and would be identified as a mark intensity of four and a complexity of one. A fish with a thermal mark designation of 1,6H would have undergone one sequence of one cycle, then another sequence of six cycles prior to hatching, giving it an intensity of seven and a complexity of two. Additionally, we quantified mark duration as the number of hours that the fish was subjected to temperature fluctuations from initial temperature spike until the final return to ambient temperature signaling the end of the thermal marking process, not including the hiatus between pre- and post-hatch sequences found in the longest mark. Sample sizes of groups, brood year and collection years of specific marks used in comparisons are reported in Table 1, and visual representations of two thermal marks are provided in Fig. 1.

To quantify the occurrence of vaterite, otoliths were soaked in deionized water for 5–10 min then placed in a black petri dish under a dissecting microscope for examination. Projected overhead light and double-polarizing filters were used to maximize contrast between vaterite and aragonite. Normal aragonite appeared opaque and white, whereas vaterite was semi-transparent and was more jagged around the otolith edges (Fig. 5). In contrast to previous studies (Sweeting et al., 2004), we dichotomously categorized otoliths as either aragonitic (normal) or vateritic (crystalline), given that the majority of otoliths were either purely aragonite or mostly vaterite (Table 1). Because no directional difference in vaterite formation was observed, the right otolith was used unless it was lost or damaged, in which case the left otolith was used instead. Quantifying the extent of thermal marking's influence on developmental disruption was conducted by comparing vaterite rates between (1) differing mark intensities, (2) mark complexity, and (3) the amount of time it took to apply a mark.

We assessed the relationship between vaterite frequency and straying by comparing Hph individuals collected from release locations where they were determined to have correctly homed (DIPAC raceway and adjacent Salmon Creek) to Hps individuals that had entered Sawmill Creek during the summer of 2015. By doing so, our sampling design controlled for brood stock and brood year effects. Samples assessed for differences in vaterite frequency and mark type came from DIPAC and SSRAA and were collected from either the hatchery raceway or terminal harvest area release location.

Frequencies of vaterite occurrence of Hph (collected at DIPAC) and Hps groups (collected at Sawmill Creek), as well as between Hps and Np groups (collected from Sawmill Creek) were tested using two-tailed Z-Score tests for two population proportions to determine whether significant differences existed between the groups. Logistic regressions were used with a specified binomial distribution to determine if frequency of vaterite occurrence increased with increasing thermal mark intensity, complexity, or duration. Only otoliths supplied by SSRAA were used for this comparison. These fish were collected as adults at release locations and otoliths were binned based on mark type (Table 1).

### Analysis of cortisol

To compare cortisol concentrations between Np, Hps and Hph individuals, we targeted chum salmon that had recently entered Sawmill and Salmon creeks and captured them using dip nets or hook and line. Targeted fish were seen entering freshwater or determined to have entered within a most 48 h based lack of scar, fungus, or fin erosion diagnostic of spawning activity and instream life. Fish were anesthetized using a 35 ppm dose of Aqui-S 20E (Aqui-S, New Zealand Ltd). Blood samples were collected within 5 min of initiating a chase to minimize bias caused by chasing/handling stress (Kubokawa et al., 1999). Each fish was tagged with a unique disc tag, revived and released back into Sawmill Creek. Instream lifespan and egg retention are described in McConnell et al. (2018). Briefly, lifespan was calculated by counting the days between tagging and recovery, and egg retention was calculated by enumerating remaining eggs within an un-scavenged body cavity and converting to a percentage based on a fecundity/length relationship (see McConnell et al., 2018). Otoliths removed from tagged carcasses from Sawmill Creek were used to associate cortisol concentration to origin through presence or absence of thermal marks. Otoliths from tagged and untagged carcasses were also used to determine the frequency of vaterite occurrence for Np and Hps groups. The Hph individuals were sampled for cortisol concentrations in Salmon Creek using identical methods to those in Sawmill Creek. Cortisol samples were collected from Hph chum returning to Salmon Creek rather than the fish ladder at the hatchery because Salmon Creek more accurately represents natural stream conditions and is closer to conditions experienced by fish captured in Sawmill Creek.

Plasma cortisol concentrations were assayed using enzyme immunoassays (EIA) obtained from Enzo Life Sciences (Farmingdale, NY, USA) and processed following Atkinson (2015).

### Testing validity of plasma cortisol concentrations

All EIA were analyzed using a Chromate microplate reader (Awareness Technologies, Palm City, FL, USA) utilizing a 405 nm filter and mass was calculated using a four-parameter logistic curve. For validations, pools for each sex were serially diluted (range: undiluted to 1: 128) in the appropriate assay buffer supplied by the manufacturer and dilutions were run as samples according to manufacturer's instructions. EIA were prepared by diluting neat serum in the appropriate assay buffer to 1:200 and to avoid inter-assay variability all samples were run in duplicate within an individual tray of assays. Accuracy of each EIA for both sexes was determined by addition of a standard material to the pools of each sex and comparison through regression of the mass added to the pool versus the mass measured for each assay. Serial dilutions for both sexes exhibited displacement parallel to that of the standard curve. The accuracy check resulted in slopes of y=−12.11+1.00×; r^2^=0.99 and y=−5.20+0.99×; r^2^=0.99 for males and females, respectively. Percent recovery of added standard was 98.72% (±5.43 s.d.; 5.51% CV) for males and 98.33% (±1.93 s.d.; 1.97% CV) for females.

Visual examination of cortisol concentration distributions revealed evidence of right-skewness and non-normality and thus data were log-transformed prior to formal statistical analysis. Exploratory analyses were conducted to identify potential outliers and verify that other confounding effects were not biasing data. These included checking for differences in cortisol concentrations based on capture technique, between up- and downstream capture locations in Sawmill Creek and testing for relationships between cortisol concentration and duration of sample collection and freshwater entrance timing (i.e. through time by day of year), as well as temperature, dissolved oxygen and salmon density, which could elicit acute stress responses, masking basal cortisol concentrations (Table 2). This allowed generalized linear models (GLM) to be used to test the effects of origin and homing location in relation to cortisol concentrations upon freshwater entry for both sexes (sex included as categorical factor to models). Instream lifespan and egg retention rates (response variables) to cortisol concentrations upon freshwater entry (predictor variable) were also compared using GLMs on transformed data. Distributions were specified as Gaussian for lifespan comparisons and binomial for egg retention rates. Samples used for lifespan and egg retention comparisons were restricted to fish deemed to have died of natural causes (senescence) because their lifespans were more likely affected by internal physiological processes rather than external forces such as bear or bird predation. To be clear, cortisol concentrations used for instream life analyses were only compared among chum salmon that authors were confident had recently entered freshwater, as evidenced by presence of sea lice, shiny coloration, lack of fin wear and location of capture. Recapture and/or re-sighting of tagged fish confirmed rates at which salmon incurred wear or changed coloration. Mean and range of cortisol concentrations are reported in the text and in figures as untransformed ng/ml for ease of comparison to other papers reporting concentrations of salmon species.

All statistical analyses were conducted in R (version 3.2.2).
